# Effects of lactose content in milk replacer on apparent digestibility, growth, liver messenger RNA expression, and blood parameters related to metabolism of dairy calves

**DOI:** 10.3168/jdsc.2023-0528

**Published:** 2024-05-10

**Authors:** R. Fukumori, M. Hirose, I. Norimura, T. Nakayama, K. Shimada, H. Mineo, M.A. Steele, S. Gondaira, H. Higuchi, K. Chisato, S. Oikawa, K. Izumi

**Affiliations:** 1 Department of Veterinary Medicine, School of Veterinary Medicine, Rakuno Gakuen University, Ebetsu, Japan 069-8501; 2 Department of Sustainable Agriculture, College of Agriculture, Food and Environment Sciences, Rakuno Gakuen University, Ebetsu, Japan 069-8501; 3 The National Federation of Dairy Co-operative Associations (Zen-Raku-Ren), Shinjuku, Tokyo, Japan 969-0223; 4 Department of Animal and Bioscience, University of Guelph, Guelph, Ontario, Canada, N1G2W1; 5 Department of Health and Nutrition, Hokkaido Bunkyo University, Eniwa, Japan 069-1449

## Abstract

Even with the same energy supply, differences in energy sources may affect calf growth and metabolism. In this study, we evaluated the effects of feeding 3 different milk replacers (MR) with different lactose supplies under isoenergetic conditions on calf digestibility, growth, and metabolism-related markers. Fifteen Holstein bull calves were randomly assigned to one of 3 MR feeding treatments: low lactose (L: 38%), medium lactose (M: 41%), or high lactose (H: 46%). After birth, calves were provided colostrum, and treatment MR were fed from 1 d of age and gradually increased to a maximum feeding rate at 20 d of age (L: 1.16 kg/d, M: 1.21 kg/d, H: 1.26 kg/d DM) without feeding solid feeds during the experimental period. Blood samples were temporarily collected weekly to assess blood concentrations of metabolites and hormones. From 30 to 36 d of age, the calves were euthanized and liver samples were collected to determine growth-related mRNA expression. The L calves showed a greater body length than H calves and the highest growth hormone receptor (GHR) mRNA expression. Plasma concentrations of total cholesterol, urea nitrogen, total protein, albumin, insulin, and IGF-1 were not different, but plasma concentrations of triglycerides were greater in order H, M, and L. These results showed that the difference in lactose content in the MR affected calf metabolism, and the L-MR was suggested to be more likely to enhance growth into the peripheral tissues.

## Highlights

• Fat/lactose ratio in MR did not change weight gain but changed tissue distribution.
• Hepatic gene expression of growth hormone receptor was lower in calves with high milk lactose.
• High-lactose milk goes more toward gastrointestinal development than peripheral tissue growth.

## Graphical Abstract

**Figure:**
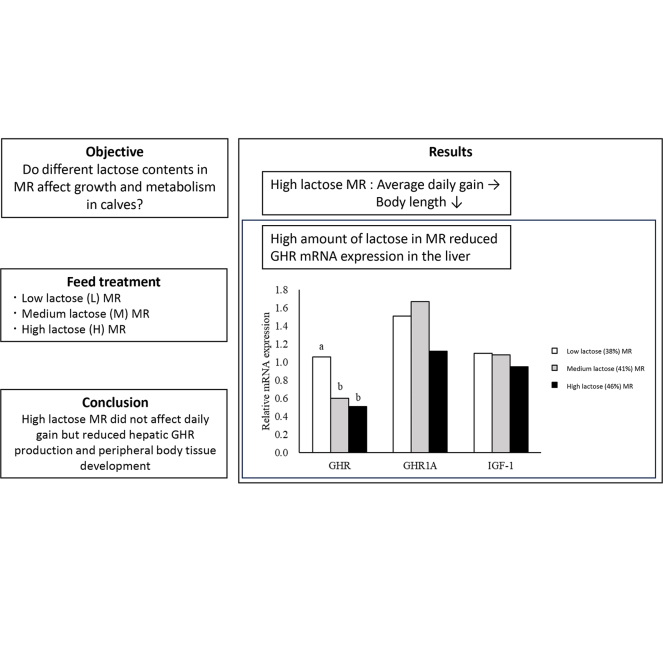
**Summary:** This study evaluated the effects of lactose content in milk replacements on the growth performance, digestibility, and metabolism of calves under iso-osmotic and isocaloric conditions. Low-lactose milk replacer (MR) did not affect average daily gain and showed greater body length and growth hormone receptor messenger RNA expression in the liver. These results suggest that the difference in the energy source of the MR affects metabolism of calves.

Recently, researchers and the dairy industry have been increasingly pressed to improve preruminant calf nutritional management because higher preweaning growth is associated with calf health and future performance (Soberon et al., 2012). The energy sources of milk replacer (**MR**) are lactose and fat, and their ratios vary based on the product, which may affect calf growth and metabolism. In a previous study where high-fat MR and high-lactose MR were fed ad libitum (Berends et al., 2020), high-lactose MR showed greater ME intake but no impact on developmental outcomes was observed. However, no conclusions have been reached regarding the optimal balance of fat and lactose as energy sources in terms of metabolic traits as well as BW gain. Lactose, a disaccharide synthesized from glucose and galactose, accounts for more than 80% of the sugar fraction in milk (Urashima and Saito, 2005) and provides the same amount of nutrients to the intestinal tract at half the osmolality of monosaccharides, reducing the risk of diarrhea due to increased osmolality. Galactose has been reported to contribute to neonatal and early-life brain development (Cederlund et al., 2013), and various benefits are expected for calf health and growth. In addition, ruminants have lower activity of ATP-citrate lyase and NADP-malate dehydrogenase (Hanson and Ballard, 1967), indicating that extra glucose might not be easily converted to body fat, supposing high-lactose MR may prevent fat accumulation compared with high-fat MR.

Growth hormone (**GH**) and IGF-1 play important roles in body growth and mammary gland development (Schäff et al., 2016). Blood circulating IGF-1 is mainly produced in the liver and its synthesis depends on hepatic GH receptor (**GHR**) abundance (Butler et al., 2003). The mRNA expression of these factors has been reported to be associated with energy and protein intake (Haisan et al., 2018; Firmenich et al., 2020), but the effect of energy sources, such as lactose content in the MR, on the GH-IGF-1 axis has not been determined. In hepatocyte studies, stimulation with glucose or insulin increases GHR expression (Dehkhoda et al., 2018). It was therefore hypothesized that for the same energy content, high-lactose MR would be more effective for development via increased GH and IGF-1 synthesis. This study aimed to determine the effects of replacement of lactose by fat in MR under conditions of equal ME supply on growth performance, blood metabolites and hormones, and growth-related mRNA expression in the liver of dairy calves.

The calves used in this study were housed at the Rakuno Gakuen University Large Animal Experiment Station (Ebetsu, Hokkaido, Japan) and all procedures were approved by the Animal Experiment Committee of Rakuno Gakuen University (approval #VH21C6; Ebetsu, Japan).

Fifteen Holstein bull calves born on 2 farms (4 and 11 calves were provided, respectively) were used in this study and assigned to one of the 3 dietary treatments with consideration given to avoid bias from farm and dam parity. The treatments consisted of MR lactose content (**L**: low-lactose MR, **M**: medium-lactose MR, and **H**: high lactose MR) for 30-d periods. Calves were fed colostrum within 3 h of birth and then transferred to the experimental location within 1 d of birth. All calves had serum IgG concentrations, measured using a bovine ELISA kit (Bethyl Laboratories, TN), above 10 mg/mL 24 h after ingestion of colostrum, with no differences between treatments (L: 24.5 mg/mL, M: 21.1 mg/mL, and H: 21.5 mg/mL; *P* = 0.84). Calves were kept in individual calf pens (2.42 m × 1 m, 2.42 m^2^/head) and fed the MR treatment 3 times a day at 0700, 1300, and 1900 h. Calves were allowed to drink water ad libitum; however, solid feed was not provided during the experiment. The lactose contents were 38%, 41%, and 46%, the CP contents were 25.2%, 25.4%, and 24.4%, and the fat contents were 17.8%, 15.8%, and 13.6% for L, M, and H, respectively. The calves were fed MR at 0.6 kg/d in L, 0.625 kg/d in M, 0.65 kg/d in H (1 to 9 d of age); at 0.9 kg/d in L, 0.938 kg/d in M, 0.975 kg/d in H (10 to 19 d of age); and at 1.2 kg/d in L, 1.25 kg/d in M, 1.3 kg/d in H (20 d of age to end of experiment) on a powder basis. No calves were found to have leftover drink. Amounts of MR were adjusted to achieve equivalent ME, and the dilution rate of each MR was adjusted to 451 mOsm/kg. The concentrations of the prepared MR were 16.7%, 15.6%, and 14.9% (wt/wt) in L, M, and H, respectively.

Blood samples were collected weekly (at 1, 7, 14, 21, and 28 d of age) before morning feeding to determine the plasma concentrations of total cholesterol (**T-Cho**), albumin, total protein (**TP**), urea nitrogen (**UN**), insulin, and IGF-1. Blood samples (5 mL) were collected in heparinized evacuated tubes (Terumo, Tokyo, Japan). The tubes were then immediately placed on ice. The tubes were then centrifuged at 1,950 × *g* for 15 min at 4°C. The harvested plasma was stored at −30°C until analysis.

At 30 to 36 d of age, the calves were euthanized and liver samples were collected. For euthanasia, xylazine (10 µL/kg BW) was used as a sedative, isosol (20 mg/kg BW) as anesthetic, and suxamethonium (0.2 mg/kg BW) as a muscle relaxant, all of which were intravenously injected. After confirmation of cardiac arrest by auscultation, the liver was excised, and samples were cut into 5 mm squares approximately 1 cm from the surface of the liver in the center of the right lobe. The samples were promptly frozen in liquid nitrogen and stored at −80°C until mRNA expression analysis.

The MR samples were sent to the Japan Food Research Laboratory (Chitose, Japan) for high-performance liquid chromatography and analyzed for lactose content, and DM, CP, and ether extracts were analyzed (AOAC International, 2000).

All analyses of plasma metabolite concentrations were performed by Sapporo Clinical Laboratory Co. (Kushiro, Hokkaido, Japan) using an automatic analyzer (Olympus AU680, Olympus Corporation, Tokyo, Japan). Sicari Kit GLU (Kanto Chemical Co., Ltd., Tokyo, Japan) was used for glucose, Quick Auto Neo BUN (Shino-Test Cooperation, Tokyo, Japan) for BUN, Albumin HRII (Fujifilm Wako Pure Chemicals Corporation, Osaka, Japan) for Albumin, Exdia XL Eiken TP (Eiken Chemical Co., Ltd., Tokyo, Japan) for TP, Determiner C TG S (Kyowa Medex, Tokyo, Japan) for triglycerol (**TG**), and Determiner C-TC S (Kyowa Medex) for T-Cho were used for measurements. Plasma concentrations of insulin and IGF-1 were measured using a time-resolved fluorescence immunoassay (competitive solid-phase immunoassay). Insulin was measured as described by Masuda et al. (2019), and IGF-1 as described by Laarman et al. (2012).

Total RNA extraction, quantification, and real-time PCR were conducted as described by Nishi et al. (2020). Total RNA extraction was conducted using TRI reagent (Sigma-Aldrich, St. Louis, MO), and DNase digestion was performed using TURBO DNA-free DNase (Thermo Fisher Scientific, Waltham, MA). Total RNA was quantified via spectrophotometry using a BioSpec-nano (Shimadzu, Kyoto, Japan). For each reaction, a parallel negative control reaction was performed in the absence of reverse transcriptase (Toyobo, Osaka, Japan) using 1 μg of RNA and analyzed by the β-actin band using PCR and 1.5% agarose gel stained with ethidium bromide, visualized on a UV transilluminator. We used the melting curve analysis to evaluate each primer pair for specificity to ascertain that only one product was amplified. The reaction was performed using a Thunderbird SYBR qPCR mix (Toyobo) and a CFX Connect Real-Time System (Bio-Rad Laboratories, Hercules, CA), per the manufacturer's instructions. Thermal cycling consisted of initial denaturation at 95°C for 5 min, followed by 40 cycles of denaturation at 95°C for 15 s, annealing at 60°C for 30 s, and extension at 72°C for 30 s. Growth hormone receptor, GHR1A, and IGF-1 as growth-related markers and GAPDH as the candidate or internal control were measured. The sequences of the primers used were the same as those described for GHR, GHR1A, and IGF-1 in Witte et al. (2019), and for GAPDH in Nishi et al. (2020).

All data are presented as LSM ± SEM. For mRNA expression in the liver, the mean of the 2^−ΔΔCt^ values of 5 calves in the L group was set to 1, after which the values for individual were represented as relative values. All data were analyzed using the fit model procedure of JMP (version 13.2.1; SAS Institute Inc., Cary, NC). The present study has sufficient power (α = 0.05, β = 0.8) to detect a 15% difference in blood parameters and 10% in mRNA expression. For variables not measured over time, such as growth performance and mRNA expression, the model included fixed effects of treatment and farm of origin, and random effects of the calf. For variables measured over time, such as plasma parameters, the model included the fixed effects of treatment, farm of origin, collection day of age as a repeated measure, their interaction, and the random effect of the calf. Farm had no effect on any variables (*P* > 0.247). Differences were considered significant at *P* < 0.05, and a tendency was declared at *P* < 0.10.

Body weight, body size, and growth during the 4-wk period are presented in Table 1. Body weight and ADG were not affected by the dietary treatments (*P* = 0.333 and *P* = 0.678, respectively). Wither height at 27 d of age in L calves was higher than H calves (L: 90.1 cm vs. H: 87.0 cm, *P* < 0.05), but this was also the case at the start (at 2 d of age; L: 84.2 cm vs. H: 80.9 cm, *P* < 0.05) and there was no difference in growth range. Body length did not differ at the start (L: 73.1 cm vs. H: 70.1 cm, *P* = 0.216), but that in L calves was longer than that in H calves at the end (L: 84.8 cm vs. H: 80.8 cm, *P* < 0.05). In the present study, lactose content did not influence ADG (L: 0.719 kg/d, M: 0.693 kg/d, and H: 0.758 kg/d, respectively, *P* = 0.678), which is consistent with the results of previous studies (Berends et al., 2020; Echeverry-Munera et al., 2021; Yohe et al., 2021). Body length of H calves was smaller than that of L calves. In contrast, a companion study by Fukumori et al. (R. Fukumori, T. Nakayama, M. Hirose, I. Norimura, K. Izumi, K. Shimada, H. Mineo, M. A. Steele, S. Gondaira, H. Higuchi, T. Watanabe [Rakuno Gakuen University, Ebetsu, Japan], H. Ueda [Rakuno Gakuen University, Ebetsu, Japan], T. Sano [Rakuno Gakuen University, Ebetsu, Japan], K. Chisato, and S. Oikawa, unpublished) showed that the gastrointestinal weight to BW ratio tended to increase with increasing lactose feeding. Therefore, it was suggested that high-lactose feeding increases the available nutrient to the gastrointestinal tissues rather than the peripheral tissues.

**Table 1:** Effects of lactose content in milk replacer (MR) on growth performance (LSM ± SEM)

Item	Treatment^1^			SEM	*P*-value
	L	M	H		
At 1 d of age					
BW (kg)	48.7	48.3	44.9	1.89	0.333
Body height (cm)	84.2^a^	82.4^ab^	80.9^b^	0.90	0.074
Hip height (cm)	86.9	85.9	85.5	1.13	0.700
Body length (cm)	73.1	71.5	70.1	1.15	0.216
Heart girth (cm)	86.8	87.4	84.0	1.78	0.383
At 27 d of age					
BW (kg)	67.4	66.3	64.6	1.55	0.473
Wither height (cm)	90.1^a^	89.4^ab^	87.0^b^	0.85	0.057
Hip height (cm)	94.0	92.6	91.2	0.98	0.183
Body length (cm)	84.8^a^	82.8^ab^	80.8^b^	1.14	0.083
Heart girth (cm)	95.8	97.2	93.6	1.40	0.225
ADG (kg/d)	0.719	0.693	0.758	0.052	0.678
Body height growth (cm)	18.7	18.0	19.7	1.34	0.678
Hip height growth (cm)	7.10	6.70	5.68	0.541	0.202
Body length growth (cm)	11.7	11.3	10.7	1.40	0.883
Heart girth growth (cm)	9.00	9.80	9.60	1.92	0.954

a,b Means within a row with different superscripts differ significantly (*P* < 0.05).

1 L = low-lactose MR (38% lactose); M = medium-lactose MR (41% lactose); H = high-lactose MR (46% lactose).

Weekly changes in the basal plasma concentrations of glucose (A), TG (B), T-Cho (C), UN (D), TP (E), albumin (F), insulin (G), and IGF-1 (H) are shown in Figure 1. Except for insulin, those profiles were changed weekly: plasma T-Cho and albumin concentrations increased weekly (Figure 1C, 1F, *P* < 0.001), but plasma UN and TP decreased (Figure 1D, *P* = 0.003; Figure 1E, *P* < 0.001). Plasma IGF-1 concentrations decreased at 7 d of age and increased again at 14 d of age (Figure 1H, *P* < 0.001). No dietary effects were observed in plasma parameters other than TG. Plasma TG concentrations were affected by dietary treatment (*P* = 0.02, Figure 1B) and were higher in order H, M, and L (*P* < 0.05). Unexpectedly, plasma glucose concentrations were not different between treatments (*P* = 0.353) and plasma TG concentrations were greater when calves fed a greater amount of lactose in MR. The reason for unchanged plasma glucose may be that the H calves had increased glucose consumption in the gastrointestinal tract due to an increased gastrointestinal tissue weight ratio, which consumes a large amount of glucose (Britton and Krehbiel, 1993). The response in plasma TG concentration to feeding MR differing fat-to-lactose ratio disagreed with previous studies. Yohe et al. (2021) reported plasma TG concentration was not changed and Berends et al. (2020) reported that plasma TG concentration was greater in calves fed high-fat MR than in those fed high-lactose MR. Adipocytes and skeletal myocytes metabolize TG as fatty acids in mitochondria for oxidation (Flier et al., 1989), but this uptake may be impaired in H calves. Further research is needed on the relationship between the fat and lactose content of the MR and body fat composition, and glucose and lipid metabolism.

**Figure 1:**
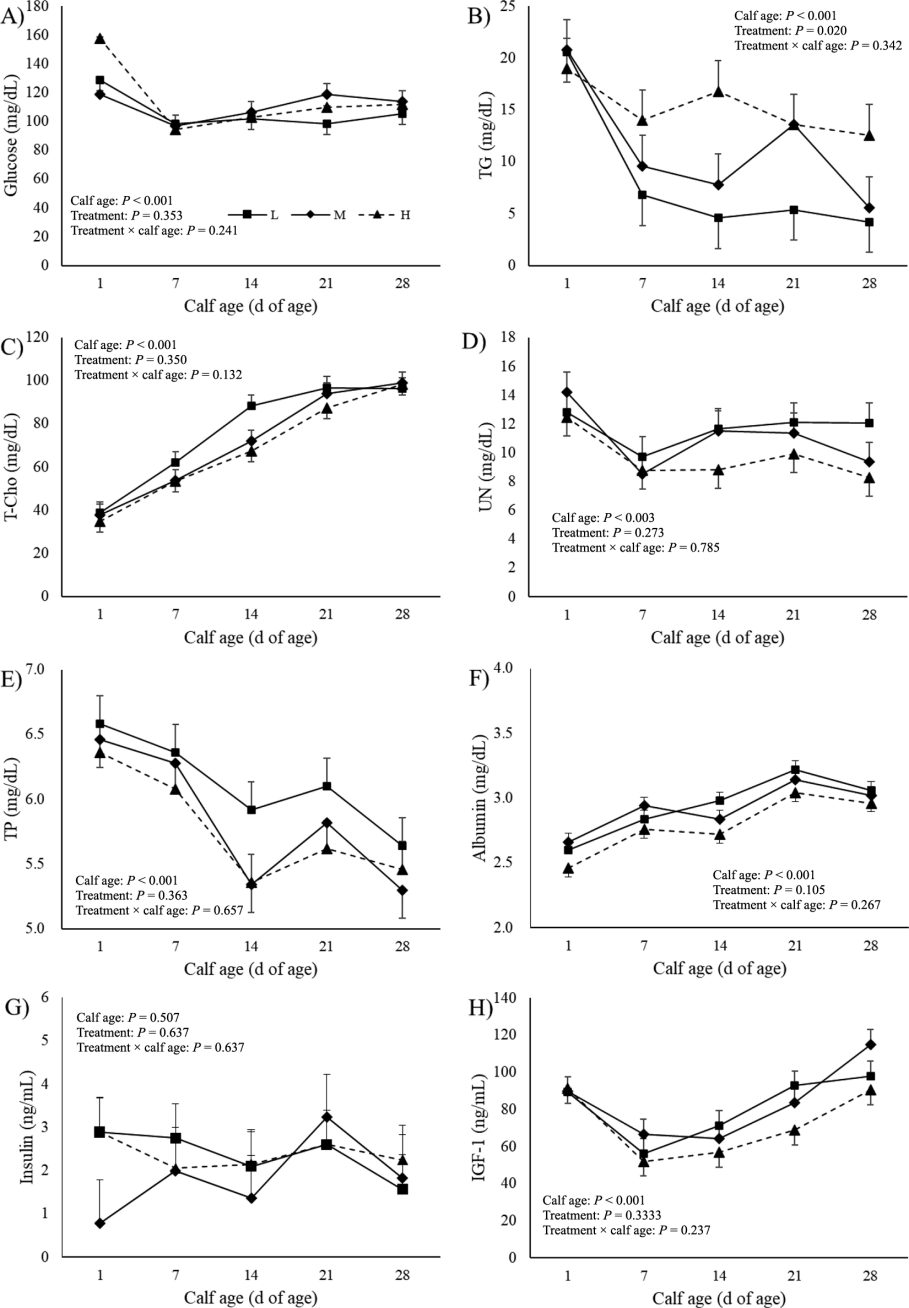
Least squares means and SEM (n = 5) for weekly changes in plasma concentrations of glucose (A), triglycerol (TG; B), total cholesterol (T-Cho; C), urea nitrogen (UN, D), total protein (TP; E), albumin (F), insulin (G), and IGF-1 (H) in calves fed milk replacer with different lactose content: low lactose (L: 38%), medium lactose (M: 41%), and high lactose (H: 46%). Plasma TG concentrations were greater in the order H, M, and L (*P* < 0.05). Means with different letters are significantly different among treatments (*P* < 0.05).

Hepatic mRNA expression is presented in Figure 2. The mRNA expression of GHR was greater in L calves than in H and M calves (*P* < 0.05). However, GHR1A and IGF-1 mRNA expressions were not different between treatment (*P* = 0.485 and *P* = 0.837, respectively). A previous study using swine cell cultures showed that increasing the concentration of glucose in the culture medium increased GHR mRNA expression in the liver (Brameld et al., 1999). In the present study, although portal glucose concentration was not measured, the H calves showed a lower postprandial rise in blood glucose concentration (in unpublished companion paper by R. Fukumori, T. Nakayama, M. Hirose, I. Norimura, K. Izumi, K. Shimada, H. Mineo, M. A. Steele, S. Gondaira, H. Higuchi, T. Watanabe [Rakuno Gakuen University, Ebetsu, Japan], H. Ueda [Rakuno Gakuen University, Ebetsu, Japan], T. Sano [Rakuno Gakuen University, Ebetsu, Japan], K. Chisato, and S. Oikawa), suggesting there might be less glucose release from visceral tissues to the liver through the portal vein. No significant differences were found in IGF-1 and GHR1A gene expression or plasma IGF-1 concentrations among the dietary treatments. The GHR1A, a specific GHR in the liver and its mRNA expression is positively correlated with IGF-1 mRNA expression (Butler et al., 2003; Kim et al., 2004), and in a previous study in young goats, IGF-1 and GHR1A mRNA expression levels were reduced when a protein-restricted diet was fed (Firmenich et al., 2020). When MR (20% fat and 30% CP) intake was increased to achieve ADG of 0.5, 0.95, and 1.4 kg/d, calf hepatic GHR1A and IGF-1 mRNA expression and plasma IGF-1 concentration increased with greater nutrient intake (Smith et al., 2002). Because the present study had an adjusted MR feeding amount for isoenergetic and isoprotein intake, there was probably no difference in these items. In addition, previous studies in rats have shown that plasma IGF-1 levels are mainly dependent on the protein content of the diet and are less influenced by carbohydrate content (Gehring et al., 2021). This suggests that the increased lactose content in the MR had little effect on IGF-1 levels in the liver and blood.

**Figure 2:**
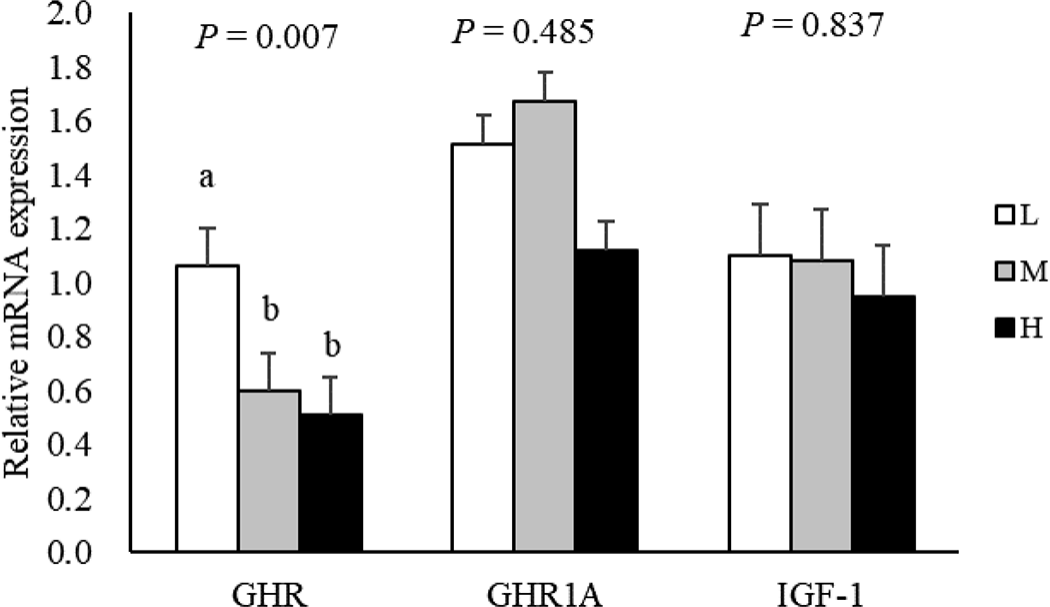
Least squares means and SEM (n = 5) for hepatic mRNA expression in growth hormone receptor (GHR), GHR1A, and IGF-1 in calves fed milk replacer with different lactose content: low lactose (L: 38%), medium lactose (M: 41%), and high lactose (H: 46%). Means with different letters are significantly different among treatments (*P* < 0.05).

Whereas previous studies have examined the ratio of lactose to fat in MR-fed solid feeds together (Berends et al., 2020; Echeverry-Munera et al., 2021; Yohe et al., 2021), the present study fed only MR to eliminate other factors. The results showed that high-lactose MR had a slightly negative effect on growth and length development. Taken together with the companion report, it was suggested that high-lactose MR may reduce the nutrient supply to peripheral tissues instead of promoting gastrointestinal development.

Other studies have reported that a high-lactose MR increases starter intake during the preweaning period, likely to compensate for energy intake (Berends et al., 2020), which may be beneficial in terms of gastrointestinal tract development for weaning. In the present study, although solid feed intake was not measured, the gut developmental effect of high-lactose MR may contribute to greater solid feed intake during the weaning period. In contrast, high lactose has been reported to have more therapeutic events than high-fat MR (Berends et al., 2020). This has also been reported in the feeding of skim milk (Reed, 1915), and low-fat intake for a few weeks may be avoided from a calf health perspective. The changes in growth factors and blood parameters in this study also support the idea that some fat content is required in the first few weeks of life for body development and health. Further functional evaluation of the fat-to-lactose ratio with respect to metabolism, such as heat production and the immune system, is necessary. In conclusion, high-lactose (46%) MR showed shorter body length and lower hepatic GHR mRNA expression than low-lactose (38%) MR, suggesting that too high of an amount of lactose may be undesirable for peripheral tissue development.
